# Two Steps Li Ion Storage Mechanism in Ruddlesden–Popper Li_2_La_2_Ti_3_O_10_

**DOI:** 10.1002/advs.202410543

**Published:** 2025-01-22

**Authors:** Mi Jang, Sunhyun Hwang, Ji Su Chae, Gun Jang, Ho Seok Park, Younki Lee, JungHyun Choi, Won‐Sub Yoon, Kwang Chul Roh

**Affiliations:** ^1^ Emerging Materials R&D Division Korea Institute of Ceramic Engineering & Technology Jinju Gyeongnam 52851 Republic of Korea; ^2^ Department of Energy Science Sungkyunkwan University Suwon 16419 Republic of Korea; ^3^ Department of Chemical Engineering Sungkyunkwan University Suwon 16419 Republic of Korea; ^4^ Department of Materials Engineering and Convergence Technology Gyeongsang National University Gyeongsangnam‐do Jinju 52828 Republic of Korea; ^5^ School of Chemical, Biological and Battery Engineering Gachon University Gyeonggi‐do Seongnam 13120 Republic of Korea; ^6^ SKKU Institute of Energy Science and Technology (SIEST) Sungkyunkwan University Suwon 16419 Republic of Korea

**Keywords:** anode materials, layered perovskite structures, Li‐ion batteries (LiBs), ruddlesden–Popper Li_2_La_2_Ti_3_O_10_(RPLLTO)

## Abstract

Innovative anode materials are essential for achieving high‐energy‐density lithium‐ion batteries (LIBs) with longer lifetimes. Thus far, only a few studies have explored the use of layered perovskite structures as LIB anode materials. In this study, the study demonstrates the performance and charge/discharge mechanism of the previously undefined Ruddlesden‐Popper Li₂La₂Ti₃O₁₀ (RPLLTO) as an anode material for LIBs. RPLLTO exhibits two unique voltage plateaus ≈0.6 and 0.4 V(vs Li/Li^+^), due to the insertion of lithium ions into different sites within its layered structure. The electrical state of Ti is analyzed using X‐ray photoelectron spectroscopy and X‐ray absorption near edge spectra, revealing a reduction from Ti⁴⁺ to Ti^2^⁺, corresponding to a capacity of 170 mAh·g⁻¹. In situ X‐ray diffraction patterns and extended X‐ray absorption fine structure spectra demonstrate the crystal structure changes during lithiation. Complementary expansion along the a/b axes and contraction along the c axis result in a volume change of only 4%. This structural stability is evidenced by an 88% capacity retention after 1000 cycles. This study successfully showcases the lithium‐ion storage capability of RPLLTO and contributes to the development of perovskite anode materials with diverse compositions and structures.

## Graphical abstract

This study demonstrates the performance of Ruddlesden‐Popper Li₂La₂Ti₃O₁₀ as an anode material for lithium‐ion batteries, achieving 170 mAh·g⁻¹ capacity with two voltage plateaus from lithium ions stored at different sites. Unique unit cell changes during lithiation ensure high structural stability, leading to 88% capacity retention after 1000 cycles, indicating long battery life and enhanced electrochemical properties.

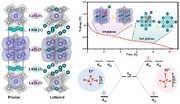

## 1 Introduction

Lithium‐ion batteries (LIBs) exhibit high energy density, high efficiency, and a long lifespan. Therefore, they have been widely used in portable batteries and electric vehicles.^[^ ^1^ ^]^ Consequently, the development of electrode materials that enhance the capacity, charging rates, lifetime, and stability of LIBs has become a crucial research area.^[^ ^2^ ^]^ Graphite, a commonly used anode material in LIBs, operates at a lower voltage of <0.1 V versus Li/Li^+^ and exhibits a specific capacity of 372 mAh·g^−1^. However, this low operating potential causes the formation of lithium dendrites, which reduce the stability of the LIBs.^[^ ^3^ ^]^ In contrast, silicon exhibits a higher operating voltage and a specific capacity of 3000 mAh·g^−1^ below 0.5 V. Nevertheless, its structural stability is compromised owing to the significant volume change caused by the incorporation of a large amount of lithium.^[^ ^2^, ^4^ ^]^ Additionally, Li_4_Ti_5_O_12_ (LTO), an oxide‐based anode material, demonstrates excellent structural stability and a capacity of 175 mAh·g^−1^ at ≈1.5 V, achieved by the redox reaction of titanium. Notably, LTO has resistance to lithium dendrite formation owing to its high operating voltage. However, its relatively low capacity limits its overall performance.^[^ ^5^ ^]^ Hence, new LIB anode materials that are resistant to lithium dendrite formation and exhibit an appropriate operating voltage, a high specific capacity, and excellent structural stability must be urgently developed.

In recent years, perovskite materials have gained widespread attention as LIB anode materials because of their unique structural and electrical properties. Zhang et al. developed perovskite‐structured Li_0.5_La_0.5_TiO_3_ (LLTO) with a specific capacity of 225 mAh·g^−1^ at a potential of <1.5 V. LLTO exhibited high lithium‐ion conductivity and had a long lifespan, which were attributed to the presence of A‐site cation deficiencies.^[^ ^6^ ^]^ Yang et al. developed double perovskite‐structured CeNb_3_O_9_ with a specific capacity of 200 mAh·g^−1^ and high rate characteristics, which were also attributed to the presence of A‐site cation deficiencies. Additionally, CeNb_3_O_9_ exhibited a high capacity and a lower average operating voltage (≈1.4 V vs Li/Li^+^) than that of LTO.^[^ ^7^ ^]^ Li et al. used SrVO_3_ as an LIB anode material and achieved an operating voltage of 0.9 V and a specific capacity of 324 mAh·g^−1^. SrVO_3_ demonstrated high structural stability, with only 2.3% volume change during the charge and discharge processes.^[^ ^8^ ^]^ Furthermore, Zhang et al. reported that La_2_MnNiO_6_ could be operated at a voltage of <0.8 V and exhibited a specific capacity of 175 mAh·g^−1^. La_2_MnNiO_6_ has a unique structure, wherein MnO6 and NiO6 have shared edges and LaO12 is arranged in the empty space.^[^ ^9^ ^]^ The above‐mentioned studies confirm the benefits of using perovskite structures as LIB anode materials, because they generally operate at an average voltage of ≈1 V and demonstrate a diverse range of capacities depending on the constituent elements and atomic arrangements.

On the other hand, the perovskite structure has the disadvantage of a high atomic packing factor (APF) due to the presence of five atoms in the unit cell. In this structure, cations occupy the A and B sites, where the A‐site cation is 12‐coordinated, and the B‐site cation is 6‐coordinated, forming a characteristic framework that contributes to its dense atomic arrangement.^[^ ^10^ ^]^ This high APF limits the empty space within the lattice, potentially reducing ionic conductivity and available lithium‐ion storage sites.^[^ ^11^ ^]^ Consequently, previous approaches to perovskite structures have aimed to increase the volume of empty space by forming layered structures or introducing defects that induce lattice distortion. Among the layered perovskite structures, the Ruddlesden‐Popper (RP) structure has a chemical formula of A_n‐1_A'_2_BnO_3n+1_, featuring an alkali metal interlayer between n perovskite layers. The ionic conductivity of this alkali metal layer can vary based on its composition and is characterized by a two‐dimensional(2D) diffusion pathway.^[^ ^12^ ^]^ Due to the interlayer, the RP structure has a lower APF compared to traditional perovskite structures, allowing for a broader ionic conduction pathway and exhibiting significant structural diversity and flexibility based on the value of n.^[^ ^13^ ^]^ Despite these advantages, there is still a lack of research on RP perovskite anodes.

In this study, we investigated the electrochemical properties and lithium insertion/extraction mechanism of a RP perovskite oxide, Li_2_La_2_Ti_3_O_10_ (RPLLTO), to determine its potential as a LIB anode material. The Li‐ion storage mechanism was elucidated by monitoring the changes in the electrochemical properties, crystal structure, and electric structure of RPLLTO during the lithiation/delithiation process.

## 2 Results and Discussion

### 2.1 Characterization of RPLLTO

**Figure 1:**
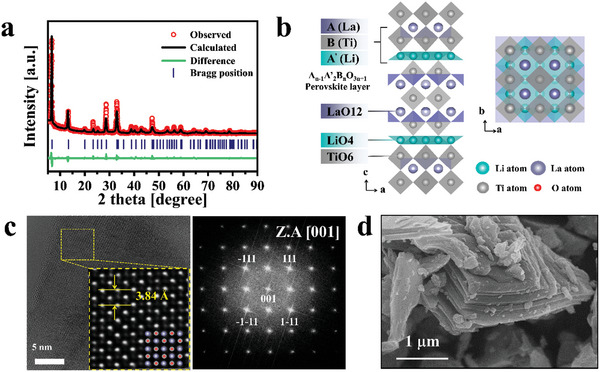
a) Rietveld refinement of the X‐ray diffraction pattern of RPLLTO powder. b) Crystal structure of RPLLTO viewed along the [100] and [001] directions. c) Large‐area high‐angle annular dark‐field image and fast fourier‐transform pattern of RPLLTO along the [001] zone axis. d) Scanning electron microscopy image of the RPLLTO particle surface at ×30k magnification.

**Figure** **1**a shows the Rietveld refinement of the X‐ray diffraction (XRD) pattern of RPLLTO. RPLLTO crystallized in the tetragonal phase and belongs to the *I*4/*mmm* space group, with lattice parameters of *a* = *b* = 3.83 Å and *c* = 26.56 Å. The refined crystal structure is shown in Figure 1b. A superlattice of three perovskite structures was stacked along the *c*‐axis. This La_2_Ti_3_O_10_ superlattice consisted of an LaO_12_ cuboctahedron and a TiO_6_ octahedron, with layers of edge‐sharing LiO_4_ tetrahedra between the superlattices.^[^ ^14^ ^]^ These interlayer structures provide a 2D Li‐ion conduction and diffusion pathway.^[^ ^12^ ^]^ Figure 1c shows a high‐angle annular dark‐field(HAADF) scanning transmission electron microscopy image. A d spacing of 3.88 Å was determined along the [001] plane. A fast fourier‐transform(FFT) pattern of RPLLTO is shown in Figure 1d. The pattern showed a distinct and consistent alignment along the *a* and *b* axes, which indicates the high crystallinity of RPLLTO. The particle shape of RPLLTO is determined via scanning electron microscopy (Figure 1e). A stepped surface and the edge site suggest the presence of various defects.^[^ ^15^ ^]^ The X‐ray photoelectron spectrum (XPS) (Figure S4, Supporting Information) of powder RPLLTO shows Li 1s, La 3d, Ti 2p, and O 1s regions. Ti^3^⁺ in the Ti 2p region and oxygen vacancy (O_v_) in the O 1s region are attributed to the broken bonds at the edge sites observed in the Figure 1d SEM image.

### 2.2 Electrochemical Properties

**Figure 2:**
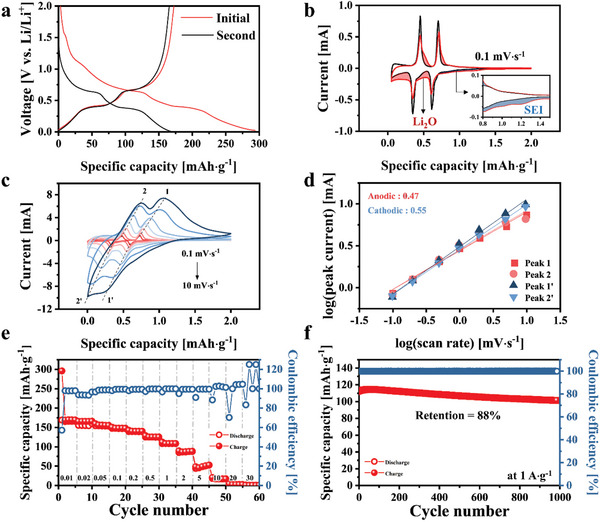
a) Galvanostatic charge/discharge profiles of RPLLTO during the initial and second cycles at a current density of 0.01 A·g^−1^ and voltage window of 0.0–2.0 V. Cyclic voltammograms of RPLLTO at a controlled scan rate of b) 0.1 mV·s^−1^ and c) 0.1–2.0 mV·s^−1^. d) log(*v*) versus log(*i*) plot in the 0.1–2.0 mV·s^−1^ range for the calculation of the *b* value. e) Rate capability in the current density range of 0.01–2.00 A·g^−1^. f) Long‐term cycling stability at 1 A·g^−1^.

The capacity and operating voltage of RPLLTO were determined using a Li‐ion half cell. **Figure** **2**a exhibits the charge and discharge profiles of RPLLTO during the initial and second cycles at a voltage of 0.0–2.0 V and current density of 0.01 A·g^−1^. RPLLTO exhibited two voltage plateaus at 0.6 and 0.4 V (Li/Li^+^). Notably, this operating voltage is lower than the average operating voltage of 1 V observed in similar perovskite structures. The operating voltage of the electrode material is determined by the number of electrons distributed in the d‐orbital of the transition metal and the bandgap. Ti has the fewest number of d‐orbital electrons (3d^0^) compared to other transition metals such as Mn (3d^5^), Fe (3d^6^), Co (3d^7^), and Ni (3d^8^), that are commonly used in battery electrode materials.^[^ ^16^ ^]^ Additionally, the bandgap of RPLLTO (2.07 eV) was smaller than that of previously reported anode materials.^[^ ^6^, ^17^ ^]^ Therefore, the low operational voltage of RPLLTO was attributed to its electronic configuration. The low operating voltage of the anode material is an advantageous characteristic in that it forms a wide potential window when a full cell is assembled, resulting in high energy density. (Figure S8, Supporting Information)

RPLLTO exhibited a capacity of ≈300 mAh·g^−1^ during the first lithiation process and a capacity loss of ≈130 mAh·g^−1^ during the delithiation process, resulting in a lithiation capacity of 170 mAh·g^−1^ during the second cycle. However, this capacity loss faded after the second cycle. The initial columbic efficiency was ≈57%, which is attributed to the irreversible capacity generated during the first lithiation process. A comparison of the first and second lithiation curves revealed irreversible capacity losses at 1.5 and 1.0 V. The capacity loss at 1.5 V is attributed to the formation of a solid electrolyte interface (SEI) layer on the electrode surface.^[^ ^18^ ^]^ In other hand, the irreversible reaction that occurred over a wide area below 1.0 V led to a larger capacity loss than that associated with SEI generation and does not fall under the voltage range of SEI formation.^[^ ^19^ ^]^

Cyclic voltammetry (CV) profiles were obtained at a specific scan rate (0.1 mV·s^−1^) and the same voltage range used for galvanostatic charge–discharge (GCD) measurement. Figure 2b shows the first and second CV curves. Similar to the GCD profile, the CV profile exhibited anodic peaks at 0.6 and 0.4 V and cathodic peaks at 0.8 and 0.5 V. This confirms the existence of a redox couple at 0.8 V/0.6 V and 0.5 V/0.4 V. Furthermore, SEI formation occurred at ≈1.5 V (Figure 2b; inner box, blue area), and Li_2_O generation occurred below 1.0 V (Figure 2b; red area).^[^ ^20^ ^]^ However, Li_2_O does not decompose during the charge process owing to its high binding energy, which leads to capacity loss.

Figure 2c shows the CV profiles of RPLLTO recorded at various scan rates ranging from 0.1 to 2.0 mV·s^−1^ within a voltage range of 0.0–2.0 V. As the scan rate increased, the current increased and the peaks broadened. This behavior can be explained by the Randles–Sevcik equation shown below:

(1) $$i_{p}=\left(2.69\times 10^{5}\right)\;n^{3/2}\mathrm{AC}\sqrt{\mathrm{Dv}}$$

where *n* is the number of electrons, *A* is the electrode area, *D* is the diffusion coefficient of the electrode material, *C* is the concentration of the electrode, and *ν* is the scanning speed. The peak current (*i_P_*) is directly proportional to the electrode concentration (*C*) and the scanning speed (*ν*), without considering the number of electrons (*n*) and diffusion coefficient (*D*). According to Equation (1), the electrode reaction of RPLLTO is restricted by mass transfer.

The charge/discharge process can be divided into a non‐Faradaic reaction, which stores electric charges on the electrode surface, and a Faradaic reaction, which stores electric charges inside the electrode. The reaction is defined by the relationship between the scan rate and current shown in the equation below:

(2) $$i_{\left(V\right)}=a\nu^{b}$$

(3) $$\log i_{\left(V\right)}=b\;log\;\nu +\log\;a$$

The slope (b value) determines if the reaction rate is controlled by the charge transfer or the mass transfer. A *b* value close to 0.5 indicates a slow electrochemical reaction occurring within the bulk of the electrode(charge transfer), whereas a *b* value near 1.0 signifies a fast reaction taking place on the electrode surface(mass transfer). Figure 2d shows a plot of log(peak current) versus log(scan rate) of each current peak extracted from the CV profile. The b value were 0.46, 0.47, 0.56, and 0.54, which are all close to 0.5. This suggests that RPLLTO demonstrates a Faradaic ion storage mechanism that mainly occurs inside the electrode rather than on the electrode surface. Other perovskites mentioned in the introduction demonstrate pseudocapacitance reactions, characterized by *b* values close to 1.0, whereas RPLLTO predominantly undergoes bulk reactions. Typically, materials that engage in bulk reactions may exhibit lower rate capabilities compared to those with pseudocapacitance.^[^ ^21^ ^]^ However, bulk‐reactive materials present certain advantages, such as lower self‐discharge rates and decreased dependence on particle size and morphology. The rate capability of RPLLTO was determined at a current density range of 0.01–30.00 A·g^−1^ (Figure 2e). The rate capability at lower currents (<2 A·g^−1^) decreased to 90% but decreased rapidly at higher currents (>5 A·g^−1^), which could be due to the dominance of slow bulk reactions in the Li‐ion storage mechanism. The RP structure allows Li ions to diffuse into the bulk due to its low APF, resulting in an extended Li diffusion path. This leads to slower ionic conductivity and, consequently, slower rate performance. In contrast, the perovskite structure exhibits a sloped GCD profile without a plateau, driven by pseudocapacitance reactions occurring on the surface, which contributes to faster rate performance.

Figure 2f shows the long‐term capacitance changes and coulombic efficiencies (CE) of RPLLTO during 1000 cycles at 1 A·g^−1^. RPLLTO demonstrated a capacity retention of 88% and CE of ≈100% after 1000 cycles. The crystal structures before and after 1000 charge and discharge cycles are compared in Figure S9 (Supporting Information). The GCD profile before and after the cycle test (Figure S9a, Supporting Information) and the XRD pattern of the electrode (Figure S9b, Supporting Information) revealed a high retention rate with no significant capacity or structural changes.

### 2.3 Charge/Discharge Mechanism

**Figure 3:**
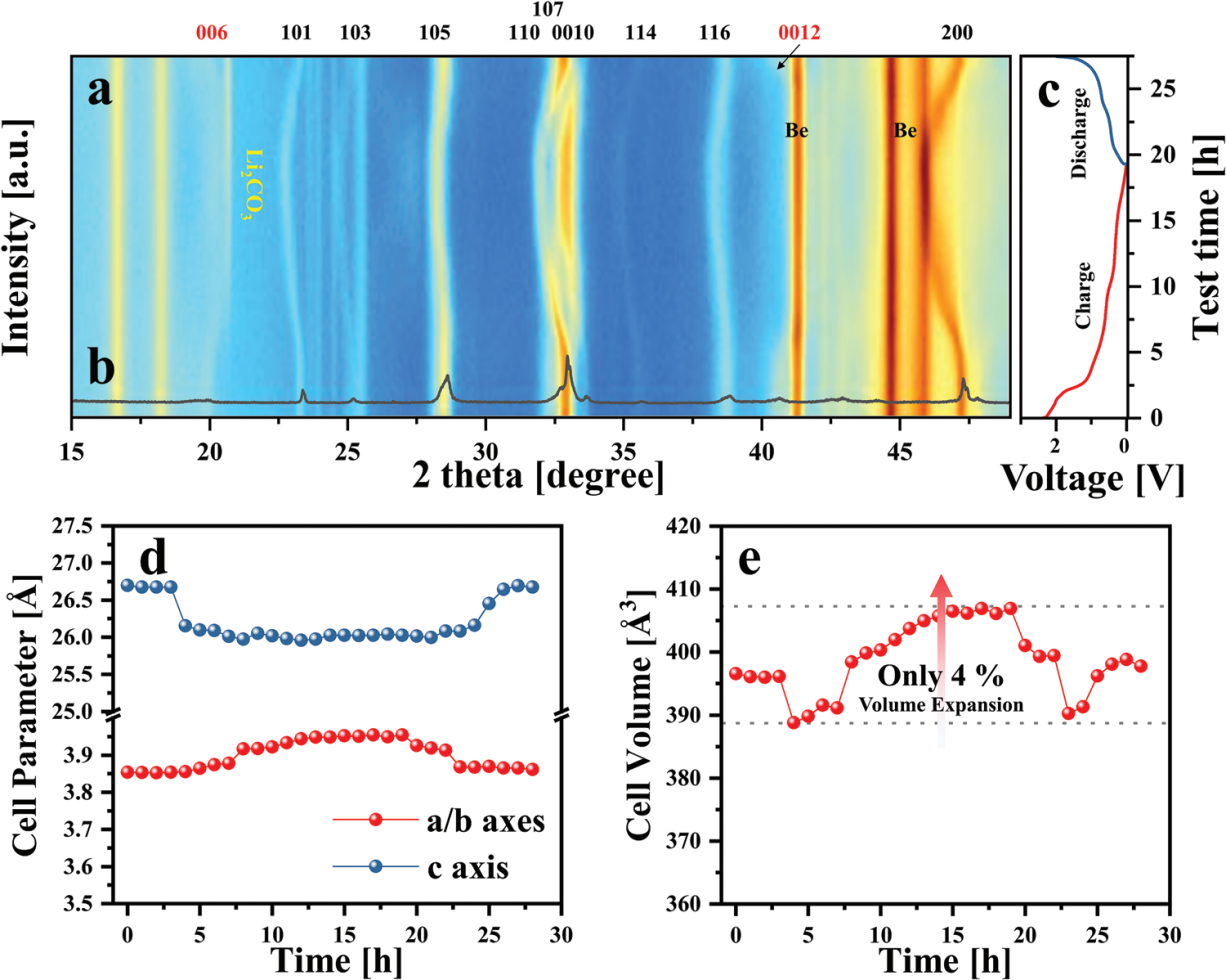
a) In situ X‐ray diffraction(XRD) pattern of RPLLTO charge/discharge at a current density of 0.01 A·g^−1^. b) Ex situ XRD pattern of the RPLLTO electrode before the test. c) Galvanostatic charge/discharge profile during in situ XRD. Changes in the d) lattice parameter and e) cell volume during lithiation/delithiation.

**Figure** **3**b shows the XRD pattern of the RPLLTO electrode, which is consistent with the in situ XRD pattern. However, some strong peaks in the latter may be attributed to the in situ XRD cell module (Figure S8a, Supporting Information). The peak shift observed in the in situ XRD patterns can be used to predict the deformation of a specific crystal plane according to Bragg's law, shown below:

(4) nλ=2dsinθ

where *n* is an integer, *λ* is the wavelength of beam, *θ* is the angle between the crystal plane and the incident beam, and *d* is the interplanar distance. If *n* and *λ* are assumed to be fixed constants, an inverse relationship exists between the measured *θ* and *d*. According to Equation (4), during the lithiation process, most of the crystal planes in RPLLTO shift toward lower angles, excluding the (006) and (0012) planes, resulting in an increase in the interplanar distance. Conversely, the (001) plane family, associated with the c‐axis, shifts toward higher angles, indicating a contraction in d‐spacing. Aside from the newly formed Li₂CO₃ peak observed near 21°, no additional peaks were detected within the measurement range. This suggests that RPLLTO experiences straightforward contraction and expansion during the lithiation/delithiation process without any phase transition. This reversible structural change was consistently observed during the second and third cycles as well (Figure S13, Supporting Information).

Ex situ XRD was performed to predict the changes in Li‐ion storage sites and interplanar distances (Figure 3e,f). The Rietveld refinement of the XRD pattern revealed an increase in the *a*/*b* axes parameter from 3.83 to 3.94 Å and a decrease in the *c* axis parameter from 26.56 to 26.03 Å. In addition, the unit cell volume of RPLLTO was increased by 4% from 389.61 Å^3^ to 404.08 Å^3^. These results indicate excellent structural stability and preservation of the crystal structure even after lithiation. Unlike layered structure anode materials such as graphite, which have a weak bonding strength along the *c* axis and expand in that direction, resulting in low structural stability, RPLLTO maintains its stability by redistributing the strain concentrated on the *c* axis to the *a*/*b* axes.^[^ ^3^, ^22^ ^]^ Moreover, the perovskite structure of RPLLTO exhibits a long cation array, which possesses low symmetry. This makes RPLLTO more flexible and suppresses phase transition. Thus, the high structural stability of RPLLTO is likely caused by the distortion of the long array of LaO_12_ and TiO_6_, which suppresses phase transition upon Li‐ion intercalation.^[^ ^13^ ^]^

**Figure 4:**
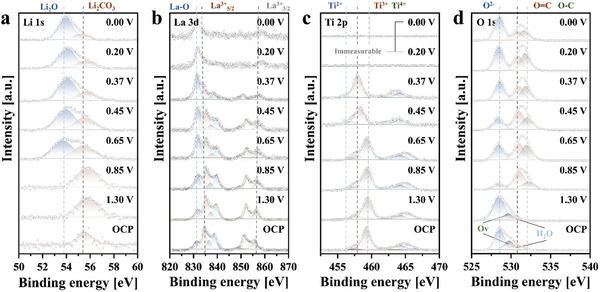
Ex situ X‐ray Photoelectron Spectroscopy during the initial lithiation by voltage state: a) Li 1s, b) La 3d, c) Ti 2p, and d) O 1s regions.

Ex situ X‐ray photoelectron spectroscopy (XPS) was performed to determine the chemical state of elements during the charge and discharge processes of RPLLTO (**Figure** **4**a–d). The Li 1s XPS revealed the presence of a Li₂CO₃ component in the OCP‐0.85 V state, which is attributed to the reaction between Li⁺ from the electrolyte and carbon, a constituent of the electrode. This is closely associated with SEI formation, as supported by the observation that the corresponding peak becomes more pronounced below 1.3 V, a voltage where SEI formation is known to occur. At lower voltages, a peak at 54 eV emerges, which can be assigned to Li₂O or Li^+^ ions. This phenomenon aligns with the Li₂O formation voltage observed in Figure 2b. The continuous increase in Li₂O concentration during lithiation suggests that Li reacts with the weakly bound oxygen distributed across the stepped surface of RPLLTO during the initial reaction, resulting in excessive irreversible capacity. Although, this may simply result from an increase in lithium concentration on the electrode surface as the voltage decreases. In the La 3d region (Figure 4b), the invariant peak positions of the La 3d₅_/_₂ and La 3d₃_/_₂ (La 3d main peaks) indicate that the oxidation state of La remains unchanged during the reaction. The satellite peak at 831 eV is associated with La‐ligand bonding. In the case of RPLLTO, the ligand involved is oxygen. As lithiation progresses, the intensity of this satellite peak changes, suggesting a modification in the electronic structure of La‐O bonding. This observation implies alterations in the electronic structure of the weakly bound oxygen mentioned earlier.

In Figure 4c, the Ti oxidation state predominantly remains +4 until the end of the first plateau, where it transitions almost entirely to +3 at 0.45 V. This indicates that all Ti⁴⁺ in RPLLTO is reduced to Ti^3^⁺ during the first reaction. The presence of a Ti^2^⁺ peak at 457 eV further suggests that a portion of Ti^3^⁺ may be reduced to Ti^2^⁺. Below 0.2 V, after the second plateau, no detectable peaks are observed in the Ti 2p region, likely due to the formation of a thick SEI layer, which prevents measurement. While the SEI comprises components such as Li₂O, LiF, Li₂CO₃, organic carbon etc., it is unlikely to incorporate transition metals, resulting in no significant signal for Ti and La. In the O 1s region (Figure 4d), the oxygen signal dynamically evolves during the reaction, reflecting the formation of various components associated with lithiation and SEI growth.

To confirm the reversibility of bonding interactions between chemical elements or changes in oxidation states during the charge/discharge process of RPLLTO, in situ Raman spectroscopy during the first lithiation and Ex situ XPS at the first and second fully lithiated/delithiated states were conducted. In situ Raman spectroscopy (Figure S10a, Supporting Information) was conducted to understand the change of bonding strength and length between elements in RPLLTO. It was conducted according to the procedure illustrated in Figure S11b (Supporting Information). The specific state of the measurements for each spectrum are indicated in Figure S12 (Supporting Information). A redshift was observed during the charge process, which is attributed to the expansion of the La–Ti bond owing to Li intercalation. This is an irreversible structural deformation. In addition, Li_2_O was generated at ≈0.5 V during the initial lithiation process, which is consistent with the voltage observed in the CV curve (Figure 2b) and Ex situ XPS (Figure 4a). Therefore, both the irreversible contraction of the La–Ti bond and the generation of Li_2_O during the initial lithiation process may have contributed to the significant initial irreversible capacity loss of RPLLTO.

Ex situ XPS of the RPLLTO electrode during the first and second charge/discharge cycles at fully charged and discharged states is presented in Figure S10 (Supporting Information). In the Li 1s region, peaks corresponding to Li⁺ and Li₂CO₃ were observed, consistent with the findings in Figure 4a. The intensities ratio of these two peaks changes reversibly and repeatedly during the charge/discharge process. The increase in the intensity of the Li⁺ peak at 0 V is attributed to the rise in Li⁺ concentration on the electrode surface and the formation of Li‐O bonds, as previously discussed. During the Li+ ion conduction process, the structural stability of these may collapse, and some may decompose, which may decrease the intensity of the related peak. The decrease in the Li^+^ peak and the increase in the intensity of Li_2_CO_3_ at 2 V prove that the concentration of Li^+^ ions on the surface decreases, and the SEI generated in the first reaction is not completely decomposed and remains during the subsequent charge process. The O1s XPS confirmed the generation of Li_2_O at ≈528 eV after the first lithiation process. This Li_2_O peak either increased or decreased during the charge/discharge cycles and did not disappear completely. These results are consistent with those of in situ Raman spectroscopy. Moreover, the peaks associated with O_v_s were observed near 530 eV and shifted slightly to the right after the first lithiation process. O_v_s have a positive charge and can accommodate electrons. Xiao et al. developed an LTO with O_v_s and analyzed its charge/discharge mechanism. They found that the O_v_s stabilized the oxidation state of Ti by accepting additional electrons, which could lead to increased capacity and improved structural stability. Similarly, as expected, RPLLTO contained O_v_s on its surface, which led to accepted additional electrons. This stabilized bond did not return to its original state after the first cycle and showed irreversible deformation.

**Figure 5:**
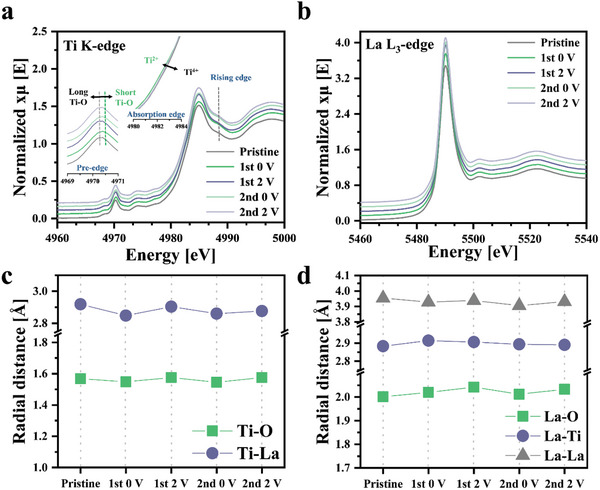
Ex situ X‐ray absorption fine structure spectra of the fully lithiated and delithiated states of RPLLTO during the first and second cycles. a) Ti K‐edge and b) La L_3_‐edge X‐ray absorption near edge spectra of the fully lithiated and delithiated states. c) Ti K‐edge and d) La L_3_‐edge extended X‐ray absorption fine structure spectra of Ti in fully charged and discharged states.

Ti K‐edge and La L_3_‐edge ex situ X‐ray absorption fine structure (XAFS) spectroscopy was performed to determine the oxidation states of the transition metals and the changes in the atomic distances during the charge/discharge processes of RPLLTO. The Ex situ XAFS spectra of the fully lithiated and delithiated states of RPLLTO during the first and second cycles are shown in **Figure** **5**. The characteristic peaks are observed in the pre‐edge, absorption edge, and rising edge regions. The pre‐edge mainly reflects the local structure and coordination environment of Ti. In particular, it can provide information about the length, coordination number, and symmetry of Ti–O bonds. The Ti K‐edge absorption spectra reveal that the pre‐edge peaks of the pristine electrode (4970.2 eV) and the electrode after the first fully lithiated state (at 0 V; 4970.4 eV) were at different positions. The rightward shift of the pre‐edge peak indicates a decrease in the Ti–O bond length. The absorption edge is directly related to the oxidation state of Ti, and a shift to the left suggests reduction, indicating a tendency toward a lower oxidation state.^[^ ^23^ ^]^ These results indicate the reduction of Ti⁴⁺ to Ti^2^⁺.^[^ ^24^ ^]^ This minute alteration in the Ti K‐edge is attributed to the strong interference of La.^[^ ^25^ ^]^ In addition, the peak at 4988.4 eV in the rising edge region was attributed to the interference between the adjacent bonds of Ti. This peak typically appears when the vibrations of adjacent bonds cause destructive interference.

Furthermore, this interference is significantly affected by the charged and discharged states and increases after lithiation. The formation of new bonds after Li intercalation confirms this increasing interference. The La L_3_‐edge absorption spectra (Figure 5b) shows negligible changes during the electrochemical process, indicating the unchanged oxidation state of La.

Figure 5c,d show the bonding distances between each element calculated from the Ti K‐edge and La L_3_‐edge Extended X‐ray absorption fine structure spectroscopy. Both O and La, which are the neighboring elements of Ti, exhibited repeated deformation before and after lithiation. This indicates the reversible Li intercalation/deintercalation around each bond, which also suggests that Li is likely stored near each bond. Except for the Ti–O and Ti–Ti bonds, most bonds undergo negligible, irregular deformation, resulting in random changes. These results indicate that RPLLTO has a highly asymmetric structure after lithiation.

**Figure 6:**
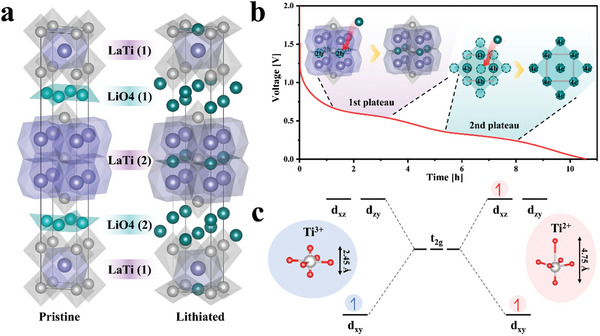
Structural analysis of RPLLTO during lithiation and delithiation. a) Crystal structures of pristine and lithiated RPLLTO. b) Changes in the crystal structure at each plateau. c) Schematic of the t_2_ _g_ orbital structure based on the Ti oxidation state.

To confirm the changes in the Li‐ion storage sites and the crystal structure of RPLLTO, ex situ XRD was conducted in the fully lithiated state (**Figure** **6**). Li was inserted into both the LiO_4_ and LaTi layers. The first plateau observed at 0.6 V could be attributed to Li storage in the LaTi layer. This is because Li exhibits the highest position stability in the O_4_ window (2b site) of the LaO_12_ cuboctahedron. Hence, we conclude that Li ions were stored at the 2b sites at the first plateau. This suggests that two sites in the LaTi(1) layer contain 1/2 Li ion each position, and four sites in the LaTi(2) layer contain a 1/4 Li ion each position. As a results, storage of two Li ions in LaTi layers. The second plateau, observed at 0.4 V, could be attributed to the LiO_4_ layer. This could be further explained as follows: when Li ions are inserted into the 4e sites of the LiO4(1) and LiO4(2) layers, these Li ions experience Li–Li repulsions with the existing Li ions in the surrounding 4d sites. The linear Li ions rearrange into a closed‐packed structure, forming a zigzag pattern and decreasing the lattice parameter along the *c* axis.

The Li intercalation process involves the rearrangement of Li and the Jahn–Teller distortion of the TiO_6_ octahedron, which changes the crystal structure of RPLLTO. The oxidation number of Ti changes from +4 to +2 (Figure 5a), during which the electron spin in the d‐orbital changes from 3d^0^ to 3d^2^. A schematic of the t_2_ _g_ orbitals (d_xy_, d_zx_, and d_yz_) and their corresponding electron spins at different oxidation numbers of Ti is shown in Figure 6c. Ti^3+^(3d^1^) is filled with electrons from the d_xy_ orbital and contracts along the *z* axis, and Ti^2+^(3d^2^) is same with Ti^3+^(3d^1^), but expands along the *z* axis.^[^ ^26^ ^]^ The relationship between this orbital transformation and the *c*‐axis deformation of the unit cell is as follows: i) the contraction of the *c* axis in the first plateau is attributed to the presence of the d_xy_ orbital of Ti; and ii) the preservation of the *c* axis length during the second plateau could be attributed to the balance between the expansion of TiO_6_ and the contraction caused by the rearrangement of Li. These minimal volume changes in the crystal structure of RPLLTO during lithiation are unique and reflect improved durability.

Based on the analysis of the crystal structure in Figure 3 and the observed electrochemical properties in Figure 2 and electronic structure changes during lithiation and delithiation in Figure 6c, we propose the following plausible Li‐ion storage mechanism of RPLLTO:

1. Initial open circuit potential at 1.0 V: SEI formation (40mAh·g^−1^)
2. Initial stage at 1.0–0.6 V: Li_2_La_2_Ti_3_O_10‐δ_ + 2x Li^+^ + 2x e^−^ → Li_2_La_2_Ti_3_O_10_ + xLi_2_O (x < 2)
3. First plateau (0.6 V): Li_2_La_2_Ti_3_O_10_ + y Li^+^ + y e^−^ → Li_2+y_La_2_Ti_3_O_10_ (reduction of Ti^4+^ to Ti^3+^) (y < 3)
4. Second plateau (0.4 V): Li_2+y_La_2_Ti_3_O_10_ + z Li^+^ + z e^−^ → Li_2+y+z_La_2_Ti_3_O_10_ (reduction of Ti^3+^ to Ti^2+^) (z < 2)
5. Reaction during the first lithiation process: Li_2_La_2_Ti_3_O_10‐δ_ + (2x+y+z) Li^+^ → Li_2+y+z_La_2_Ti_3_O_10_ + xLi_2_O
6. End of First charge/discharge reaction: Li_2_La_2_Ti_3_O_10_ + xLi_2_O

The overall experimental results indicate that RPLLTO demonstrates a mechanism capable of storing approximately six lithium ions during the initial lithiation process. In this process, the accommodation of additional electrons by O_v_, the formation of Li₂O, and reaction with a carbon material at low cutoff voltages (Figure S17, Supporting Information) contribute to the initial irreversible capacity. While the reduction of Ti ions to Ti^3^⁺ would lead to the uptake of three Li ions and a capacity of 135 mAh·g⁻¹, the partial reduction of some Ti ions to Ti^2^⁺ allows for the accommodation of additional Li ions. Therefore, our findings explain the unique electrochemical properties and charge/discharge mechanism of RPLLTO. This study will promote the design and development of LIB anode materials with various perovskite structures and compositions.

## 3 Conclusion

In this study, we investigated the electrochemical properties and Li‐ion storage mechanism of RPLLTO to validate its applicability as an anode material for LIBs. RPLLTO, synthesized via an ion‐exchange reaction, exhibited a capacity of 170 mAh·g⁻¹, which is ≈25% higher than its theoretical capacity(135mAh·g^−1^). The unique two plateaus observed at 0.6 V and 0.4V(Li/Li^+^) were attributed to lithium ions being stored at different sites. XPS and XAFS analysis revealed that while the oxidation state of Ti changes from 4+ to 2+, La does not participate in the reaction. In situ XRD and Rietveld refinement were used to track the lithium storage sites and the crystal structure changes during lithiation, confirming a only 4% volume change. This minor volume change correlates with the 88% capacity retention observed after 1000 cycles.This study will promote the design and development of LIB anode materials with different types of perovskite structures and compositions.

## 4 Experimental Section

### Materials Preparation

RPLLTO was synthesized using an ion‐exchange method. Na_2_La_2_Ti_3_O_10_ (NLTO), a precursor of RPLLTO, was synthesized through a solid‐state reaction method. Na_2_CO_3_ (99.5%, SAMCHUN, Republic of Korea) (with an excess of 20 mol% Na_2_CO_3_ to compensate for Na loss during processing), La_2_O_3_ (99.9%, Sigma Aldrich, Germany) and TiO_2_ (99.9%, Sigma Aldrich, Germany) were added to ethanol in their stoichiometric ratios and homogeneously mixed using a planetary mill (PULVERISETTE 7 Premium, FRITSCH, Germany) at 800 rpm for 10 min. The mixture was dried to a powder in an oven at 80 °C, and the powder was pelletized for calcination. The NLTO pellet was calcined at 1100 °C for 10 h and ground using a mortar and pestle for the ion‐exchange reaction.

For the ion‐exchange reaction, NLTO and LiNO_3_ were mixed using a mortar and pestle and heated at 300 °C for 12 h. To remove any remaining LiNO_3_, the mixture was washed with distilled water several times and dried in an oven at 60 °C.

### Materials Characterization

Powder XRD patterns (D8 Advance diffractometer, Bruker, Germany) of RPLLTO were acquired in the 2θ range of 5°–90° to analyze its crystal structure. The particle morphology of RPLLTO was determined using a SEM (JSM‐7600F, JEOL, Japan) at various magnifications. The atomic arrangement and interplanar distances were investigated using a STEM (Titan Cubed Themis G3 300, FEI Inc, USA) in the HAADF mode. XPS (PHI50000, FEI Inc, USA) was performed to analyze the chemical composition and oxidation state of RPLLTO.

### Electrochemical Properties

RPLLTO electrodes were fabricated using a slurry casting method. A slurry of RPLLTO, super P (Imerys S.A, France), and the carboxymethyl cellulose(CMC) (LANDT, USA) binder in an 8:1:1 ratio was coated on a Cu foil current collector using a doctor blade and dried in a vacuum oven at 110 °C for 12 h. The RPLLTO electrode was assembled into a CR2032 half cell using Li metal as the counter electrode. Polypropylene was used as the separator, and 1 M LiPF_6_ in EC/DMC (1:1 v/v) was used as the electrolyte. All electrochemical characterization was performed in the voltage range of 0.0–2.0 V versus Li/Li^+^. Constant current charge/discharge performance was performed using a galvanostat/potentiostat battery test system (WMPG1000S, WonAtech, Republic of Korea), and the current density was in the range of 0.01–2.00 A·g^−1^. CV was performed using a potentiostat (VSP, Biologic, France) at different scan rates in the range of 0.1–2.0 mV·s^−1^.

### Li‐ion Storage Mechanism

The working electrode for in situ XRD was prepared by coating a slurry of RPLLTO and a polyvinylidene fluoride binder(PVDF) on a Be sheet. During the in situ XRD measurement, the cells with the Be sheet electrode were subjected to a current density of 0.01 A·g^−1^ in the voltage range of 0.0–2.0 V versus Li/Li^+^. In situ XRD patterns were obtained using the same procedure used for powder XRD patterns.

## Conflict of Interest

The authors declare no conflict of interest.

## Supporting information



Supporting Information

## Data Availability

Research data are not shared.

## References

[1] a) K.‐N. Kang , H. Lee , J. Kim , M.‐J. Kwak , H. Y. Jeong , G. Kim , J.‐H. Jang , ACS Energy Lett. 2020, 5, 3828;

[2] a) P. U. Nzereogu , A. D. Omah , F. I. Ezema , E. I. Iwuoha , A. C. Nwanya , Appl. Surface Sci. Adv. 2022, 9, 100233;

[3] a) H. Zhang , Y. Yang , D. Ren , L. Wang , X. He , Energy Storage Materials 2021, 36, 147;

[4] L. de Biasi , B. Schwarz , T. Brezesinski , P. Hartmann , J. Janek , H. Ehrenberg , Adv. Mater. 2019, 31, 1900985. 10.1002/adma.201900985 31012176

[5] a) G.‐N. Zhu , H.‐J. Liu , J.‐H. Zhuang , C.‐X. Wang , Y.‐G. Wang , Y.‐Y. Xia , Energy Environ. Sci. 2011, 4, 4016;

[6] L. Zhang , X. Zhang , G. Tian , Q. Zhang , M. Knapp , H. Ehrenberg , G. Chen , Z. Shen , G. Yang , L. Gu , Nat. Commun. 2020, 11, 3490. 32661230 10.1038/s41467-020-17233-1 PMC7359355

[7] L. Yang , X. Xiong , G. Liang , X. Li , C. Wang , W. You , X. Zhao , X. Liu , R. Che , Adv. Mater. 2022, 34, 2200914. 10.1002/adma.202200914 35231949

[8] X. Li , Z. Lin , N. Jin , X. Yang , Y. Du , L. Lei , P. Rozier , P. Simon , Y. Liu , Adv. Mater. 2022, 34, 2107262. 10.1002/adma.202107262 34677908

[9] J. T. Mefford , W. G. Hardin , S. Dai , K. P. Johnston , K. J. Stevenson , Nat. Mater. 2014, 13, 726. 24880729 10.1038/nmat4000

[10] Q. Tao , P. Xu , M. Li , W. Lu , npj Comput. Mater. 2021, 7, 23.

[11] Q. A. Akkerman , L. Manna , ACS Energy Lett. 2020, 5, 604. 33344766 10.1021/acsenergylett.0c00039 PMC7739487

[12] S. J. Fanah , F. Ramezanipour , J. Phys. Chem. C 2021, 125, 3689.

[13] P. Ding , W. Li , H. Zhao , C. Wu , L. Zhao , B. Dong , S. Wang , J. Phys.: Mater. 2021, 4, 022002.

[14] K. Toda , J. Watanabe , M. Sato , Mater. Res. Bull. 1996, 31, 1427.

[15] A. Mathieson , M. Rahil , Y. Zhang , W. M. Dose , J. T. Lee , F. Deschler , S. Ahmad , M. De Volder , Mater. Adv. 2021, 2, 3370.

[16] J.‐K. Park , in Principles and Applications of Lithium Secondary Batteries, John Wiley & Sons, Hoboken, NJ 2012.

[17] M. G. Vergniory , L. Elcoro , C. Felser , N. Regnault , B. A. Bernevig , Z. Wang , Nature 2019, 566, 480. 30814710 10.1038/s41586-019-0954-4

[18] Y. Surace , D. Leanza , Ł. Kondracki , M. Mirolo , C. A. F. Vaz , M. El Kazzi , P. Novák , S. Trabesinger , Energy Storage Mater. 2021, 44, 156.

[19] A. Wang , S. Kadam , H. Li , S. Shi , Y. Qi , npj Comput. Mater. 2018, 4, 15.

[20] Y. Lu , L. Yu , X. W. D. Lou , Chem 2018, 4, 972.

[21] T. Kim , W. Choi , H.‐C. Shin , J.‐Y. Choi , J. M. Kim , M.‐S. Park , W.‐S. Yoon , J. Electrochem. Sci. Technol. 2020, 11, 14.

[22] S. Lee , J. H. Kim , Y.‐S. Lee , J. Im , J. Appl. Electrochem. 2021, 51, 1407.

[23] a) N. Jiang , D. Su , J. C. H. Spence , Phys. Rev. B 2007, 76, 214117;

[24] Y. Liu , Q. Bai , A. M. Nolan , Y. Zhou , Y. Wang , Y. Mo , Y. Xia , Nano Energy 2019, 66, 104094.

[25] M. Nakayama , T. Usui , Y. Uchimoto , M. Wakihara , M. Yamamoto , J. Phys. Chem. B 2005, 109, 4135. 16851474 10.1021/jp046062j

[26] B. Wen , Q. Hao , W.‐J. Yin , L. Zhang , Z. Wang , T. Wang , C. Zhou , A. Selloni , X. Yang , L.‐M. Liu , Phys. Chem. Chem. Phys. 2018, 20, 17658. 29931014 10.1039/c8cp02648c

