# Supplementary material for: Two Steps Li Ion Storage Mechanism in Ruddlesden–Popper Li2La2Ti3O10
Source: Adv Sci (Weinh). 2025 Jan 22;12(21):2410543. doi: 10.1002/advs.202410543 (PMC12140317; doi:10.1002/advs.202410543)
Supplement: Supplementary file 1 — Supporting Information [file ADVS-12-2410543-s001.docx]

Supporting Information

Two steps Li Ion Storage Mechanism in Ruddlesden-Popper Li_2_La_2_Ti_3_O_10_

Mi Jang^a†^, Sunhyun Hwang^b†^, Ji Su Chae^a†^, Gun Jang^d^, Ho Seok Park^d^, Yunki Lee^e^, JungHyun Choi^f^, Won-Sub Yoon^b,c*^, and Kwang Chul Roh^a,*^

*^a^* M. Jang, J. S. Chae, K. C. Roh

Emerging Materials R&D Division, Korea Institute of Ceramic Engineering & Technology, 52851, Republic of Korea

E-mail: [rkc@kicet.re.kr](mailto:rkc@kicet.re.kr); Tel: +82-055-792-2625; Fax: +82-055-792-2625

*^b^* S. Hwang, W.-S. Yoon

Department of Energy Science, Sungkyunkwan University, 16419, Republic of Korea

*^c^* W.-S. Yoon

SKKU Institute of Energy Science and Technology (SIEST), Sungkyunkwan University, 16419, Republic of Korea

^d^ G. Jang, H. S. Park

Department of Chemical Engineering, Sungkyunkwan University, 16419, Republic of Korea

^e^ Y. Lee

Department of Ceramic Engineering, Gyeongsang National University, 52828, Republic of Korea

*^f^* J. Choi

School of Chemical, Biological and Battery Engineering, Gachon University, 13120, Republic of Korea

^†^ These authors contributed equally to this work

| Radiation Powder X-ray (Cu Kα) λ=1.5046Å) | |
| --- | --- |
| Crystal system | Tetragonal |
| Space group | I4/mmm (No. 139) |
| Lattice parameters | a=b=3.83 Å, c=26.56 Å |
| Cell volume | 389.61 Å^3^ |
| R_wp_ | 47.3 |
| R_p_ | 44.8 |
| χ^2^ | 1.06 |

Table S1. RPLLTO Rietveld refinement result.

Table S2. Results of ICP analysis of RPLLTO.

| Atom | Weight % |
| --- | --- |
| Li | 2.33 |
| La | 46.66 |
| Ti | 24.12 |
| O | 26.2 |

| Radiation Powder X-ray (Cu Kα) λ=1.5046 Å | |
| --- | --- |
| Crystal system | Tetragonal |
| Space group | I4/mmm (No. 139) |
| Lattice parameters | a=b=3.94 Å, c=26.03 Å |
| Cell volume | 404.08 Å^3^ |
| R_wp_ | 8.56 |
| R_exp_ | 3.94 |
| S(R_wp_/R_exp_) | 2.17 |

Table S3. Rietveld refinement result of RPLLTO first lithiation at 0 V

| Radiation Powder X-ray (Cu Kα) λ=1.5046 Å | |
| --- | --- |
| Crystal system | Tetragonal |
| Space group | I4/mmm (No. 139) |
| Lattice parameters | a=b= 3.84 Å, c=26.62 Å |
| Cell volume | 392.53 Å^3^ |
| R_wp_ | 6.06 |
| R_exp_ | 3.22 |
| S(R_wp_/R_exp_) | 1.88 |

Table S4. Rietveld refinement result of RPLLTO first lithiation at 2 V





Figure S1. Scanning electron microscope image of powder RPLLTO. (a) 5k and (b) 10k.


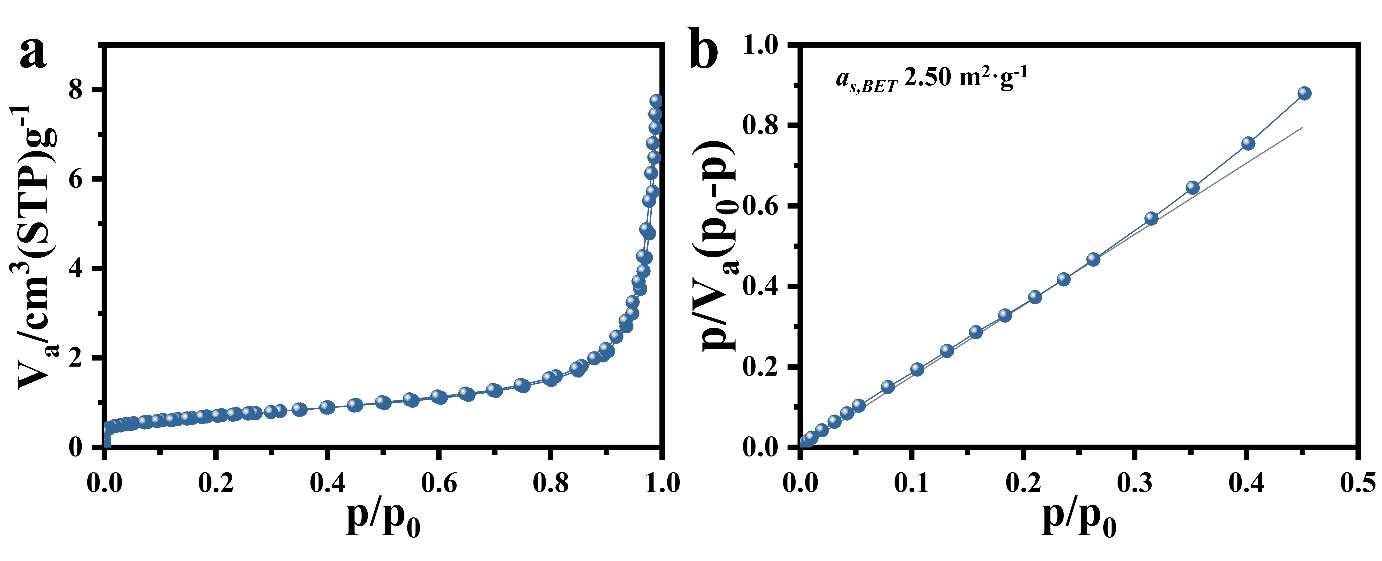


Figure S2. (a) Adsorption/desorption curve and (b) BET plot of RPLLTO.


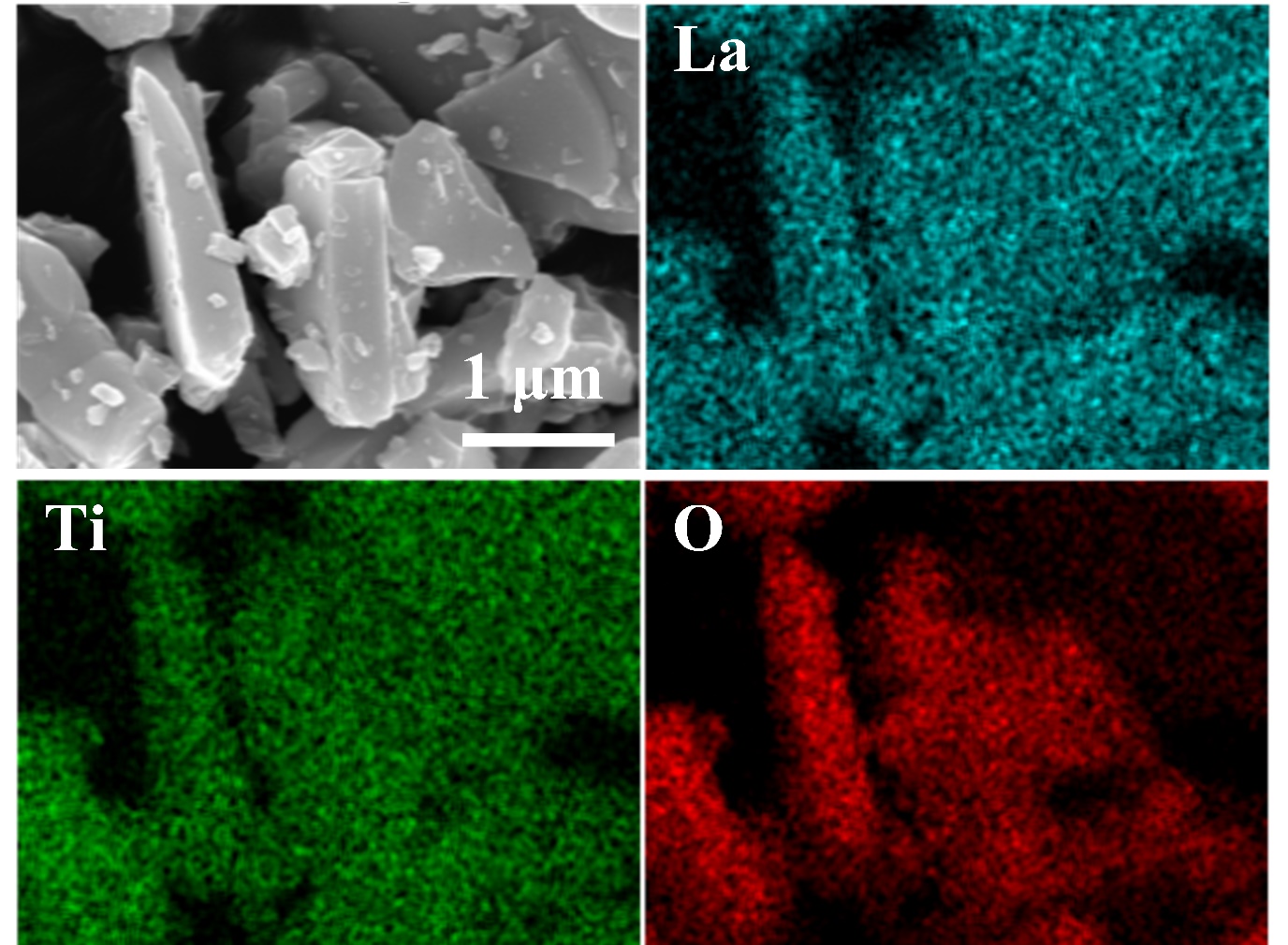


Figure S3. Energy-dispersive X-ray spectroscopy(EDS) mapping of RPLLTO.


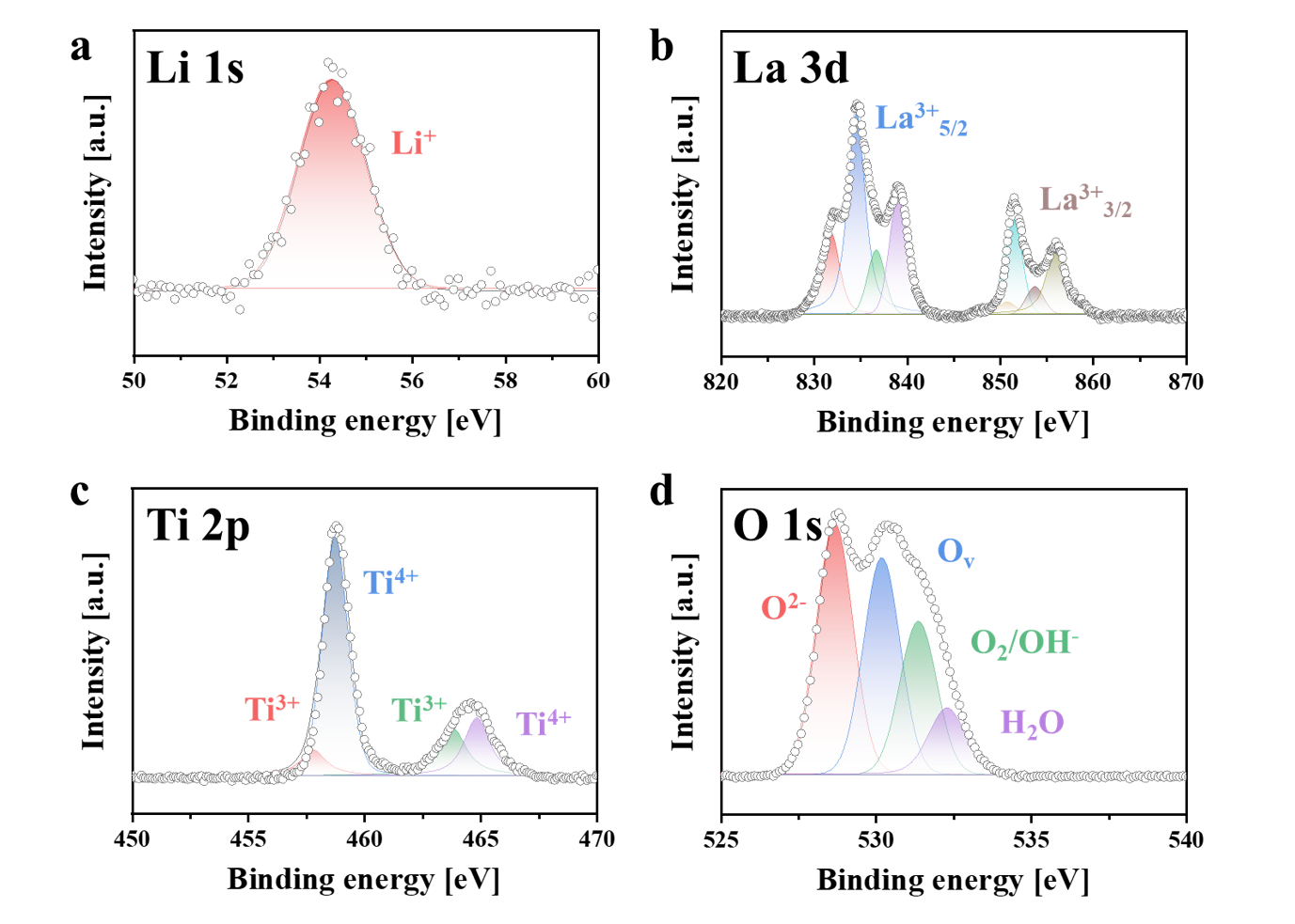


Figure S4. X-ray photoelectron spectra of powder RPLLTO. The region of (a) Li 1s, (b) La 3d, (c) Ti 2p and (d) O 1s.


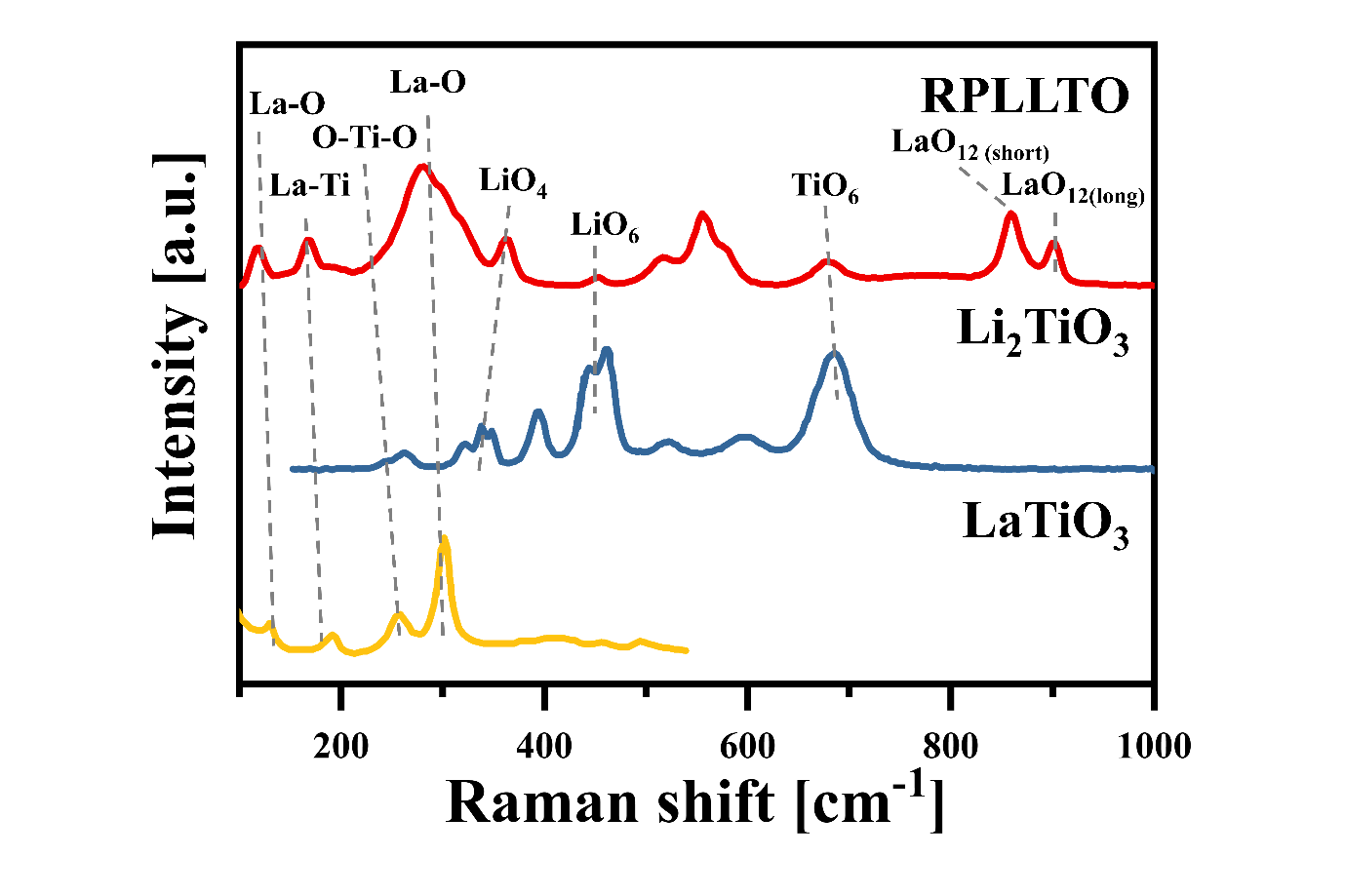
Figure S5. Raman Spectroscopy of powder RPLLTO, Li_2_TiO_3_^[1]^ and LaTiO_3_^[2]^.





Figure S6. Fourier transform-infrared Spectrometer of powder RPLLTO.


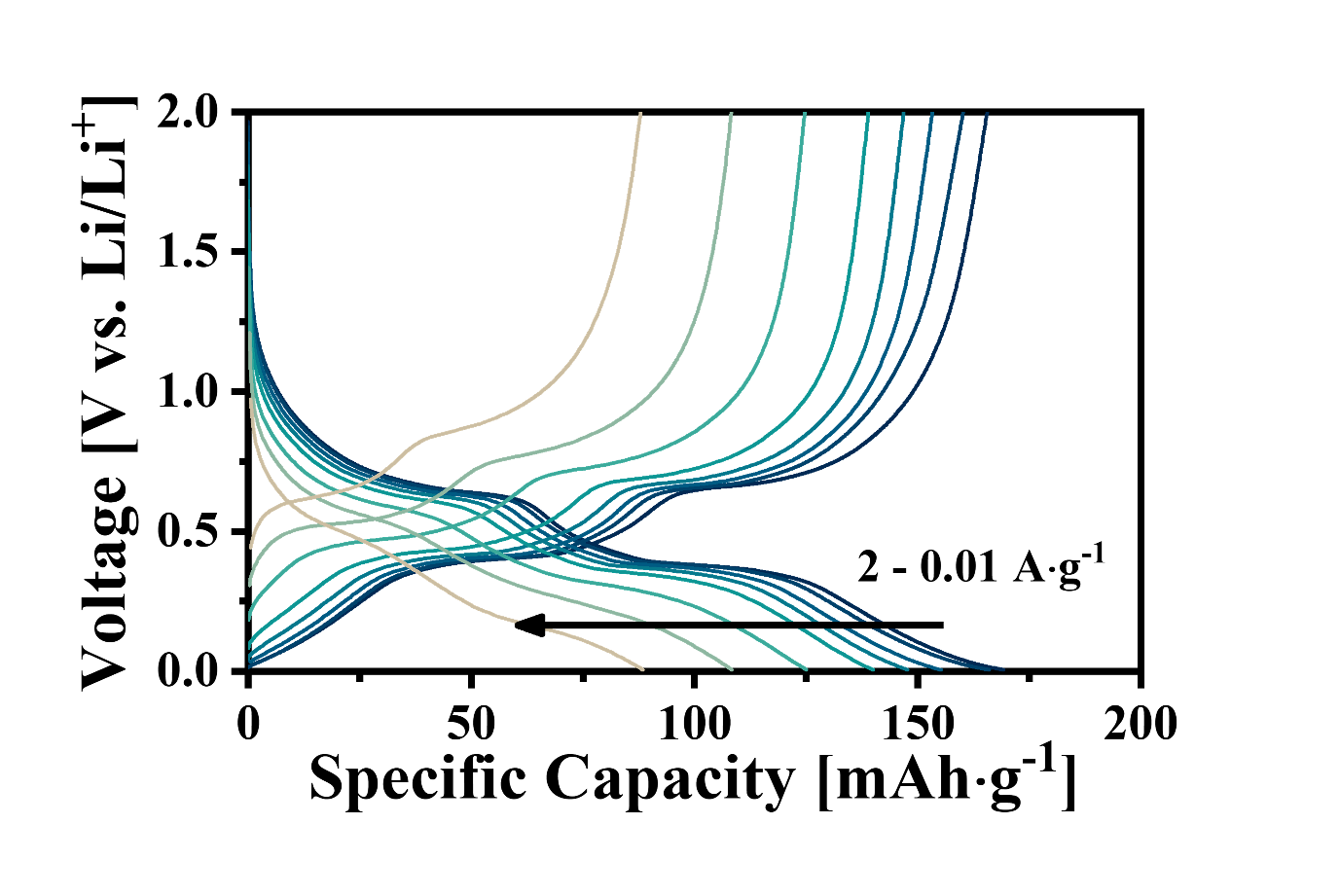


Figure S7. The galvanostatic charge-discharge curves under current density of 0.01 A·g^-1^ to 2 A·g^-1^ in the voltage window of 0 - 2 V.


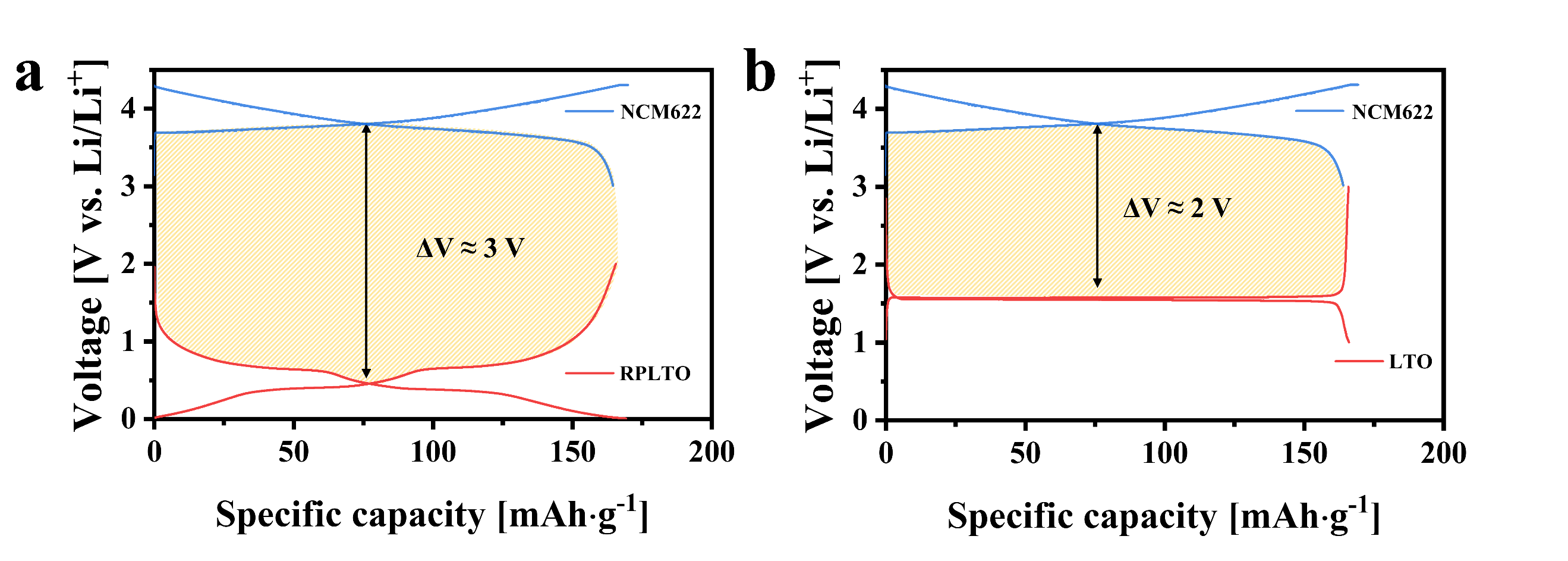
Figure S8. Average operating voltage of the full cell with NCM622 cathode material and (a) RPLLTO and (b) LTO anodes.

The NCM622 electrode (Li(Ni_0.6_Co_0.2_Mn_0.2_)O_2_, Wellcos Corporation, Republic of Korea) was assembled into a CR2032 half cell, with Li metal serving as the counter electrode. A polypropylene separator was used, and the electrolyte consisted of 1 M LiPF_6_ in a 1:1 volume ratio of ethylene carbonate (EC) and dimethyl carbonate (DMC). Electrochemical characterizations were conducted within a voltage range of 3.0–4.3 V versus Li/Li^+^. The constant current charge/discharge performance was carried out using a galvanostat/potentiostat battery test system (WMPG1000S, WonAtech, Republic of Korea), with current density 0.01 A·g⁻¹.

The electrochemical performance of RPLLTO anode may appear comparable to that of the LTO (1.5 V vs. Li/Li⁺, 175 mAh·g^-1^). However, differences can be observed when comparing the energy density of the two materials. The material density of LTO is 3.5 g·cm^-^³, while RPLLTO has a higher density of 4.92 g·cm^-^³. When comparing equal volumes, LTO provides a volumetric energy density of 612.5 mAh·cm^-^³, whereas RPLLTO offers 668.25 mAh·cm^-^³. This results in an approximate 9% difference in volumetric energy density between the two materials.

Additionally, when fabricated with NCM622 (3.5 V vs. Li/Li⁺, 170 mAh·g^-1^) as a cathode to assemble full cells, RPLLTO and LTO exhibit average operating voltages of 3.0 V and 2.0 V, respectively. Under these conditions, the full cell energy densities are calculated to be 510 Wh·kg^-1^ and 306 Wh·kg^-1^, revealing a significant 66% higher gravimetric energy density for RPLLTO full cells. This notable difference in energy density highlights the advantage of the lower operating voltage of RPLLTO over LTO, particularly in the context of full-cell energy storage.


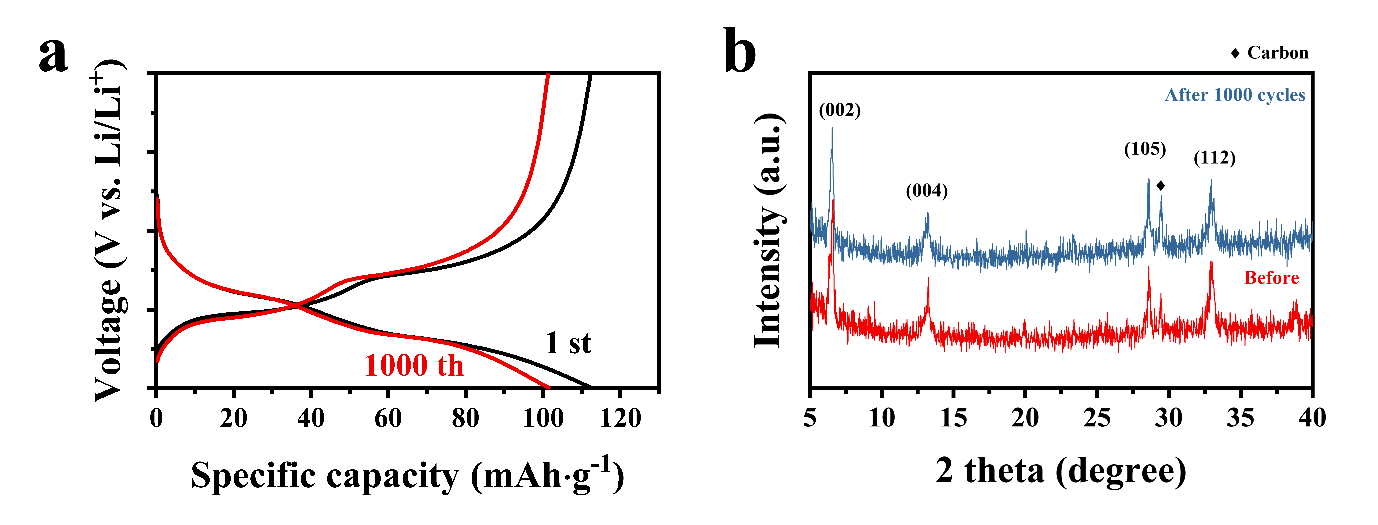


Figure S9. (a) The first and 1000th cycle GCD profiles measured at a current density of 1 A·g^-1^. (b) *Ex-situ* XRD of RPLLTO electrode in OCP state and tested for 1000th cycles.


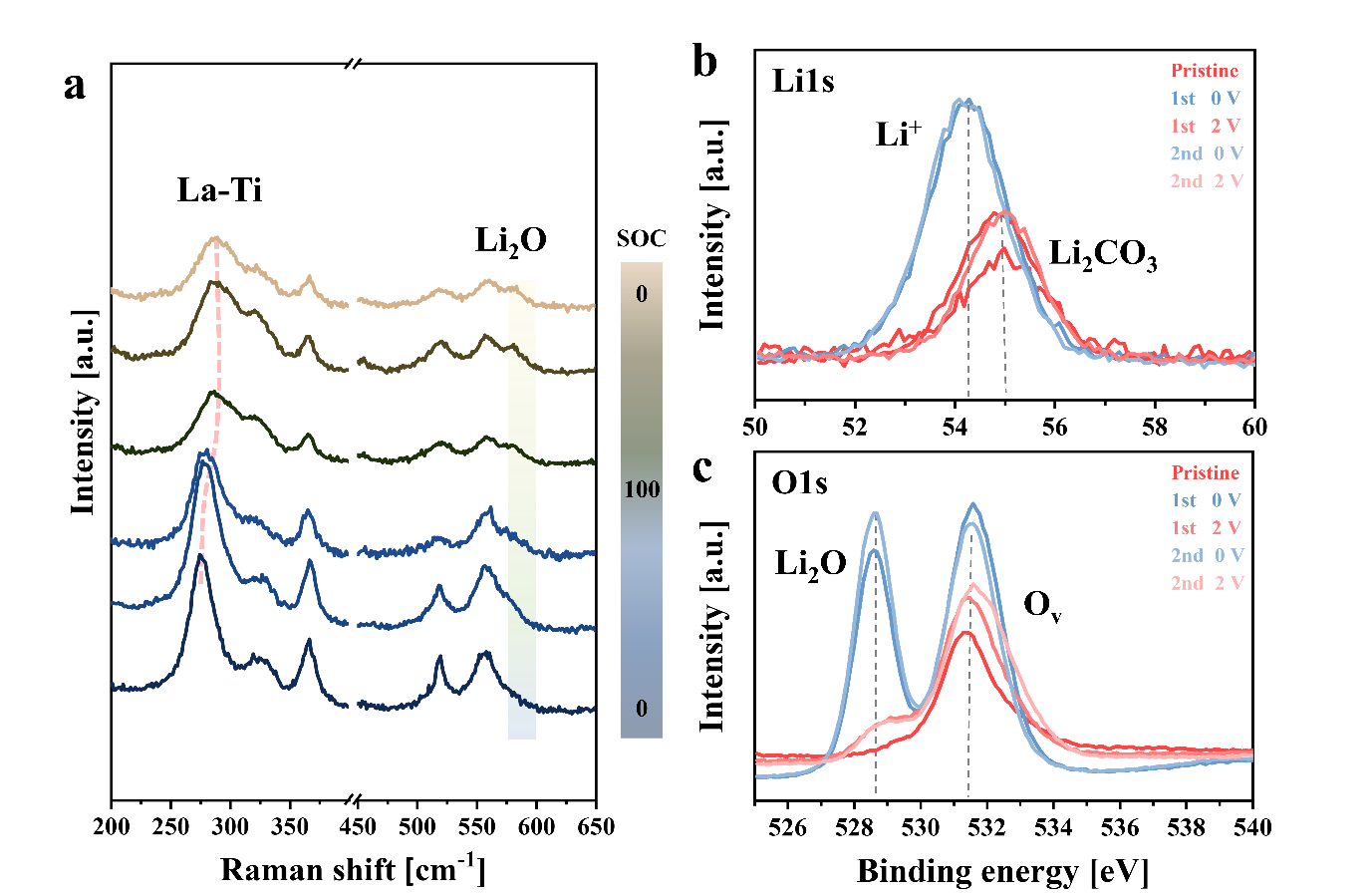


Figure S10. (a) In situ Raman spectra of RPLLTO during different states of charge (SOC). (b) Li 1s and (c) O 1s Ex-situ X-ray photoelectron spectroscopy of the lithiation/delithiation states of RPLLTO.


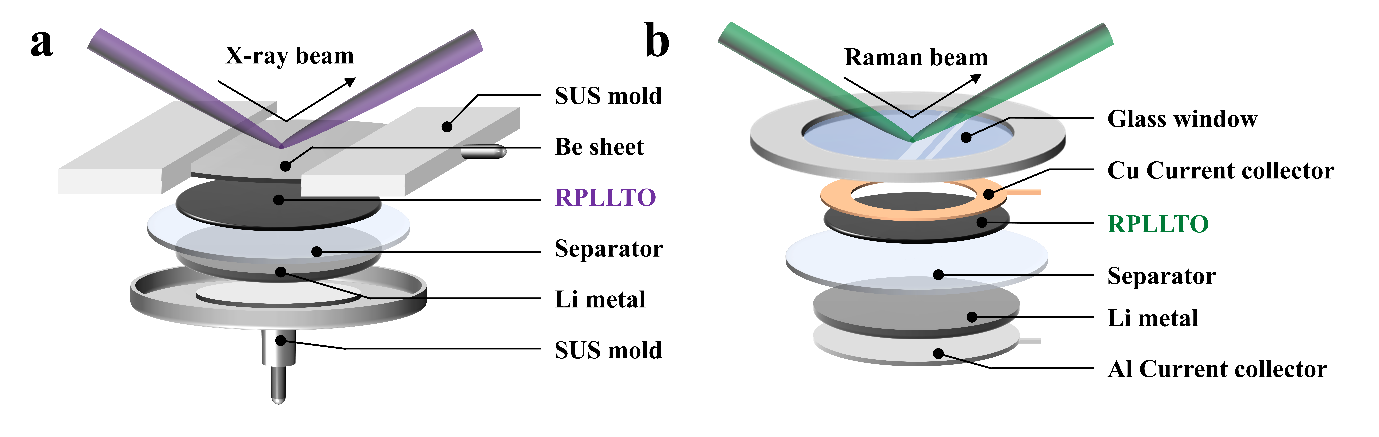


Figure S11. Schematic diagrams of the cell assembly for (a) In situ X-ray diffraction and (b) In situ Raman spectroscopy.





Figure S12. Point analyzed for *In situ* Raman spectra. Measured at a current density of 0.2 Ag^-1^.


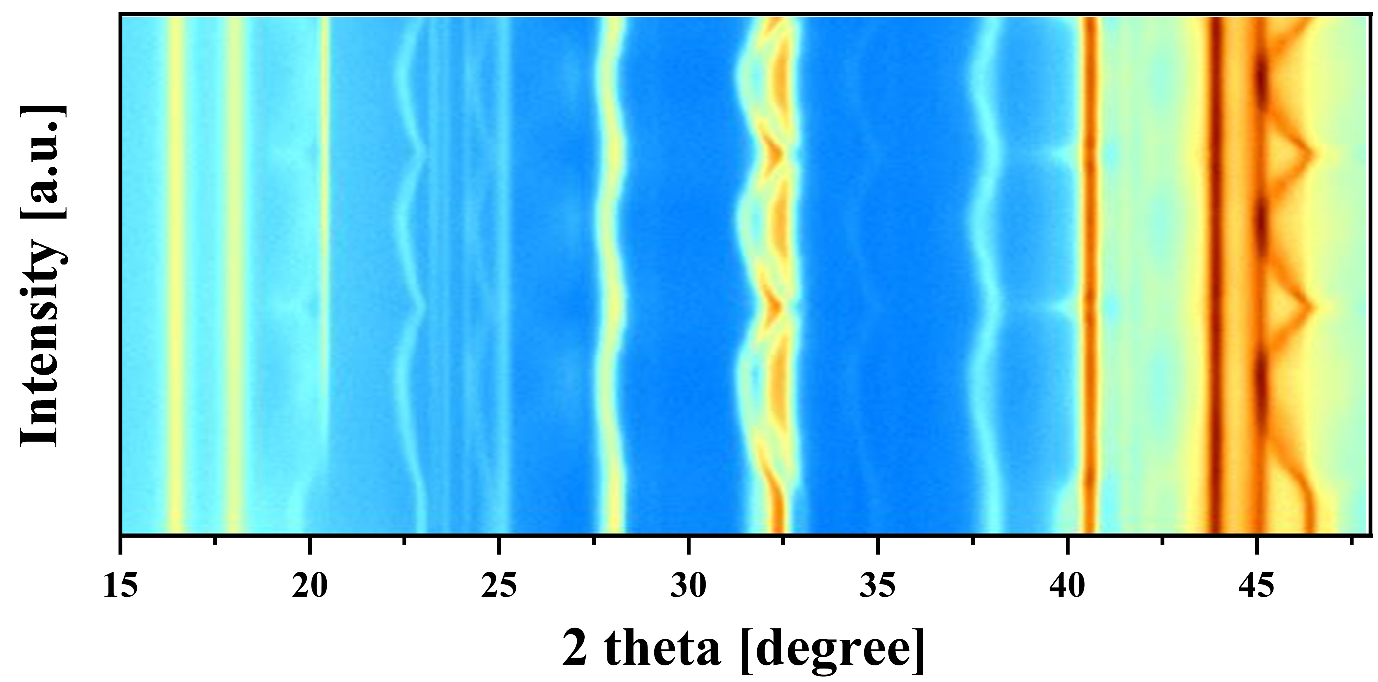


Figure S13. In situ X-ray diffraction patterns were collected during the charge/discharge of the RPLLTO electrode, which was cycled three times between 0 - 2 V under a 0.01 A·g^-1^.


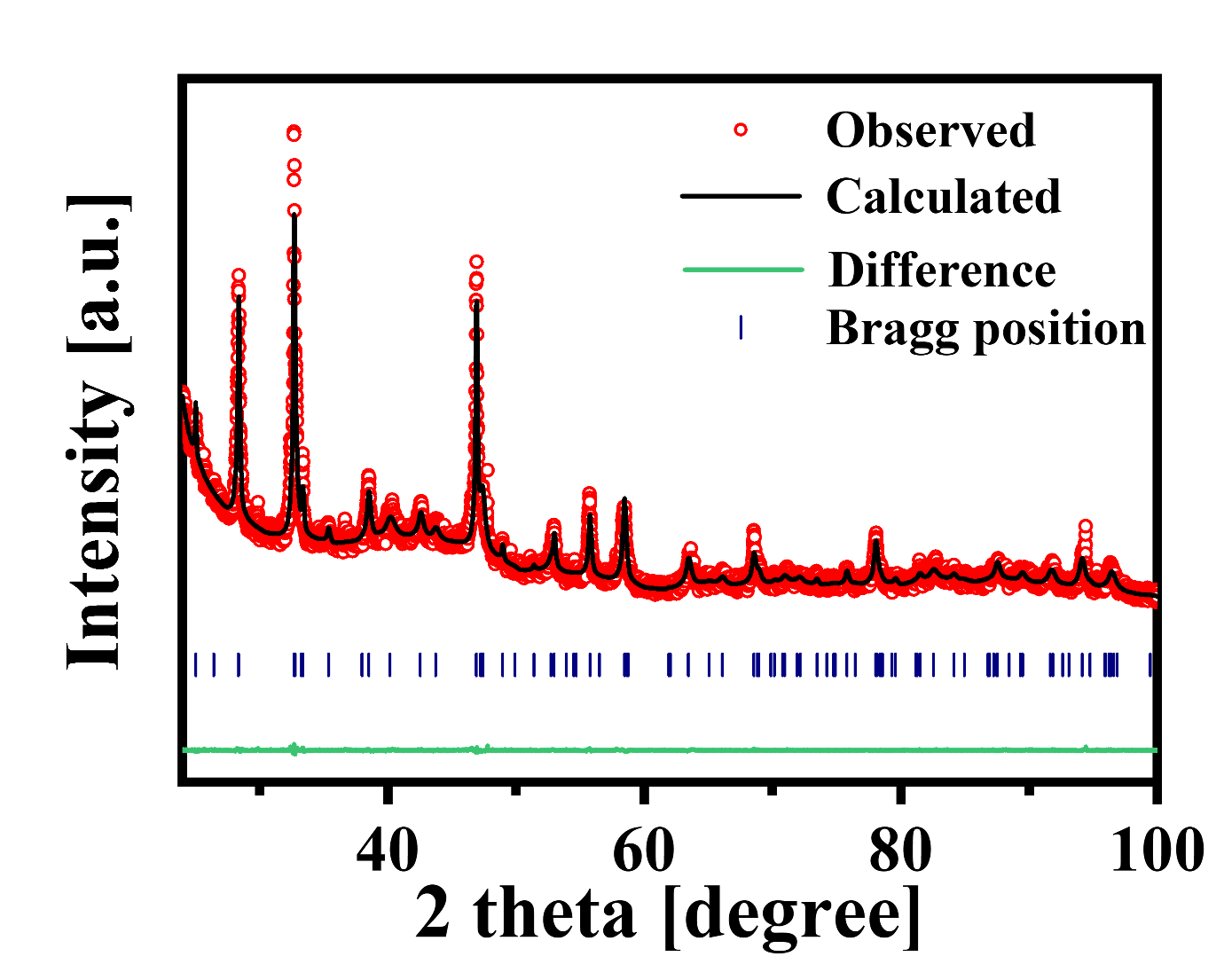


Figure S14. Ex situ X-ray diffraction Rietveld result of first lithiation (0V).


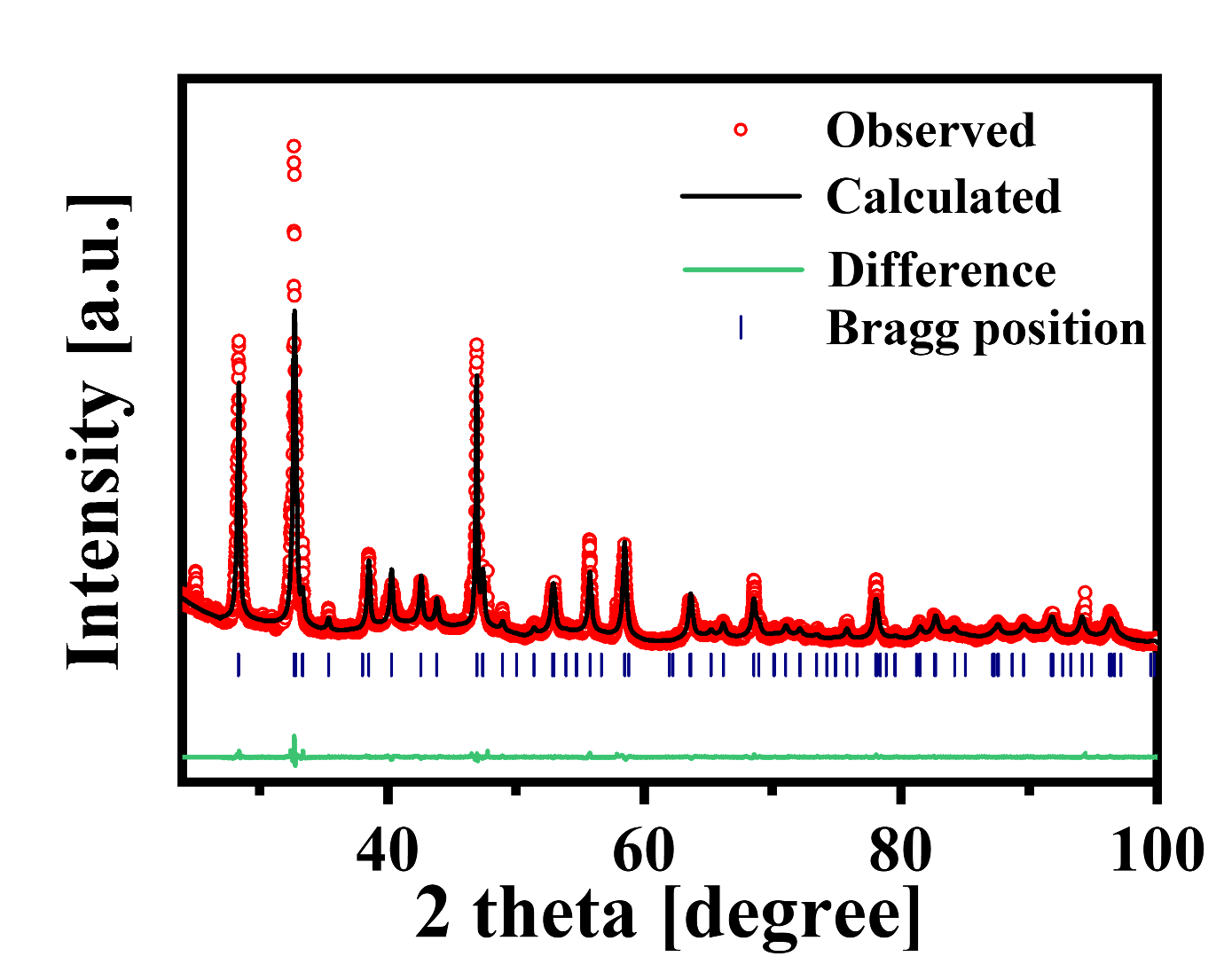


Figure S15. Ex situ X-ray diffraction Rietveld result of first delithiation (2V).


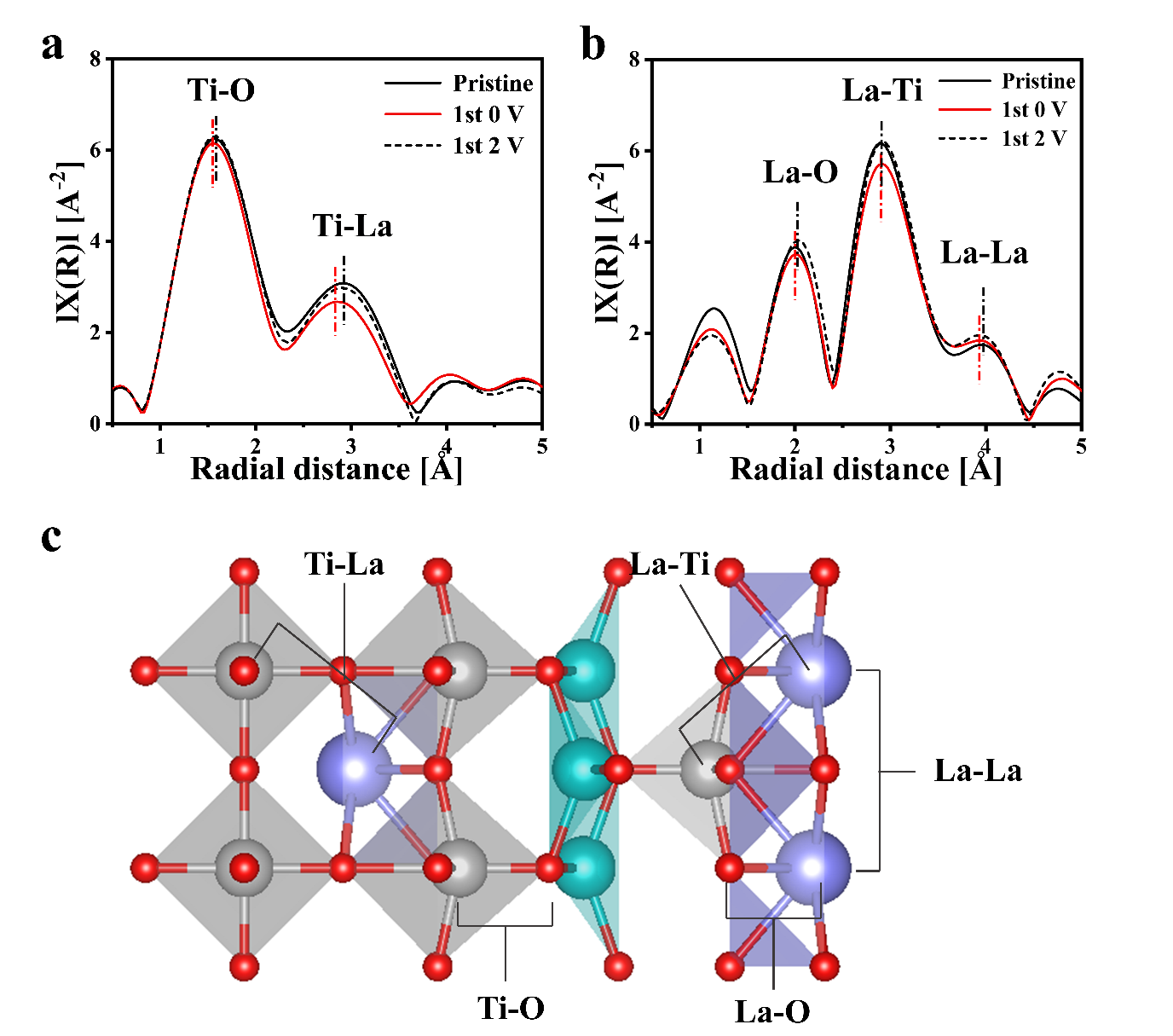


Figure S16. Changes in (a) Ti K-edge and (b) La 3L-edge Extended X-ray absorption fine structure spectra among the first lithiation (1st 0V) and delithiation (1st 2V). (c) The molecular schematic diagram and their respective bonds.


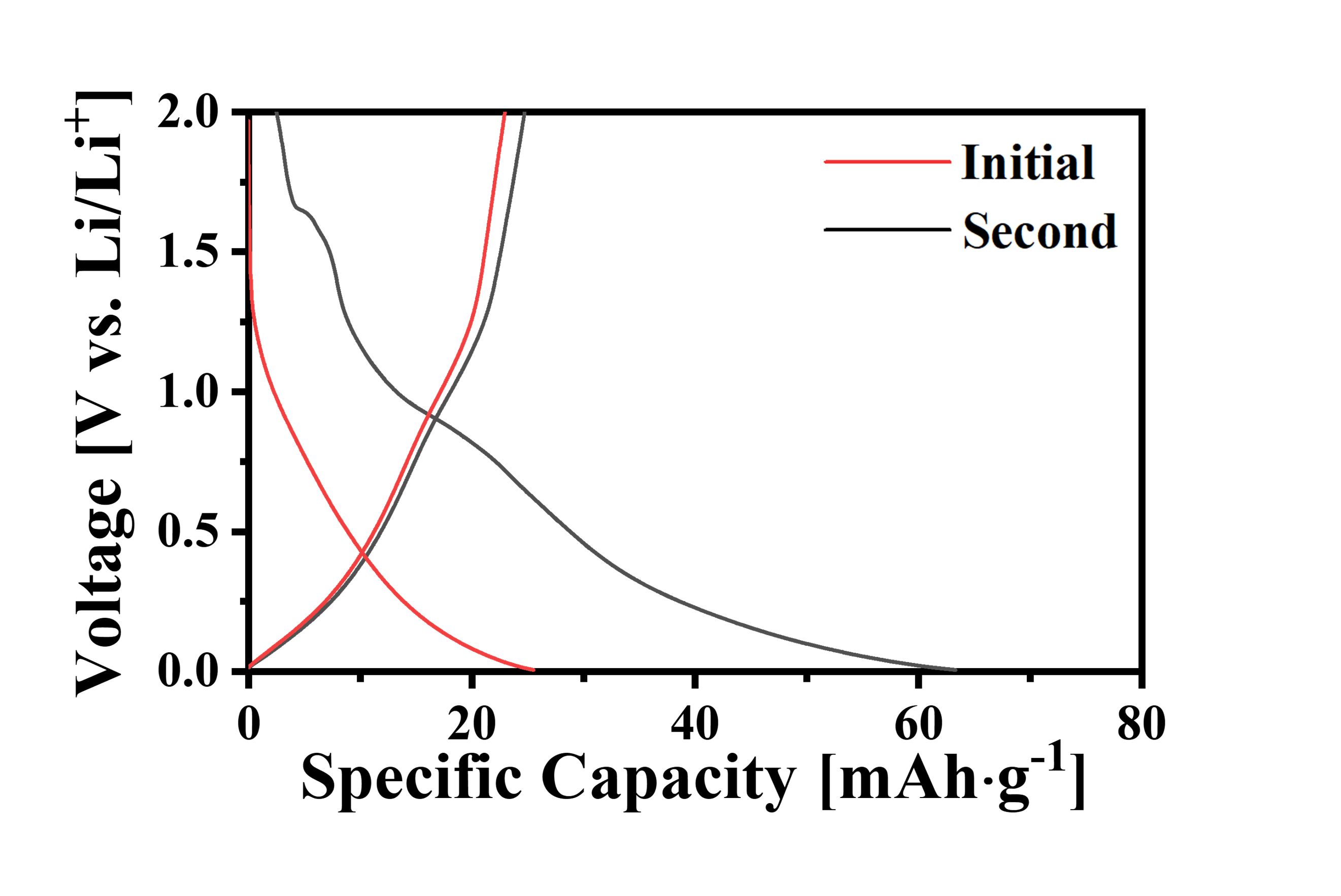


Figure S17. Galvanostat charge/discharge profile at a current density of 0.01 A g⁻¹ for a lithium ion half cell fabricated with SuperP. Super P was used as the active material and conductive agent, and CMC was used as the binder. The weight ratio of the two was calculated to be 9:1 and mixed. The measurement conditions were set the same as those of RPLLTO.

Reference

[1] A. Lakshmi Narayana, M. Dhananjaya, N. Guru Prakash, O. M. Hussain, C. M. Julien, *Ionics* **2017**, *23*, 3419-3428. <https://doi.org/10.1007/s11581-017-2147-1>.

[2] C. Ulrich, G. Khaliullin, M. Guennou, H. Roth, T. Lorenz, B. Keimer, *Physical review letters* **2015**, *115 15*, 156403.
